# Potential of Fibrin Glue and Mesenchymal Stem Cells (MSCs) to Regenerate Nerve Injuries: A Systematic Review

**DOI:** 10.3390/cells11020221

**Published:** 2022-01-10

**Authors:** Adriana de Cássia Ortiz, Simone Ortiz Moura Fideles, Karina Torres Pomini, Márcia Zilioli Bellini, Eliana de Souza Bastos Mazuqueli Pereira, Carlos Henrique Bertoni Reis, João Paulo Galletti Pilon, Miguel Ângelo de Marchi, Beatriz Flavia de Moraes Trazzi, Willian Saranholi da Silva, Marcelo Rodrigues da Cunha, Daniela Vieira Buchaim, Rogerio Leone Buchaim

**Affiliations:** 1 Department of Biological Sciences, Bauru School of Dentistry (FOB/USP), University of São Paulo, Bauru 17012-901, Brazil; adrianaortiz@usp.br (A.d.C.O.); simoneortiz@usp.br (S.O.M.F.); karinatp@usp.br (K.T.P.); dr.carloshenriquereis@usp.br (C.H.B.R.); 2 Postgraduate Program in Structural and Functional Interactions in Rehabilitation, Postgraduate Department, University of Marilia (UNIMAR), Marília 17525-902, Brazil; elianabastos@unimar.br (E.d.S.B.M.P.); danibuchaim@alumni.usp.br (D.V.B.); 3 Pro-Rectory of Research and Graduate Studies, University Center of Adamantina (UniFAI), Adamantina 17800-000, Brazil; mzbellini@fai.com.br; 4 UNIMAR Beneficent Hospital (HBU), Medical School, University of Marilia (UNIMAR), Marília 17525-160, Brazil; joao.pilon@unesp.br; 5 Postgraduate Program in Speech Therapy, Sao Paulo State University (UNESP—Univ Estadual Paulista), Marília 17525-900, Brazil; 6 Coordination of the Medical School, University Center of Adamantina (UniFAI), Adamantina 17800-000, Brazil; coordmedicina@fai.com.br; 7 Coordination of the Dentistry School, University of Marilia (UNIMAR), Marília 17525-902, Brazil; flavia.odonto@unimar.br; 8 Dentistry School, University of Marilia (UNIMAR), Marília 17525-902, Brazil; williansaranholi@unimar.br; 9 Interunit Postgraduate Program in Bioengineering (EESC/FMRP/IQSC), University of São Paulo (USP), São Carlos 13566-590, Brazil; cunhamr@hotmail.com; 10 Department of Morphology and Pathology, Jundiaí Medical School, Jundiaí 13202-550, Brazil; 11 Teaching and Research Coordination of the Medical School, University Center of Adamantina (UniFAI), Adamantina 17800-000, Brazil; 12 Graduate Program in Anatomy of Domestic and Wild Animals, Faculty of Veterinary Medicine and Animal Science, University of São Paulo (FMVZ/USP), São Paulo 05508-270, Brazil

**Keywords:** nerve regeneration, fibrin glue, fibrin sealant, scaffolds, stem cells, systematic review

## Abstract

Cell-based therapy is a promising treatment to favor tissue healing through less invasive strategies. Mesenchymal stem cells (MSCs) highlighted as potential candidates due to their angiogenic, anti-apoptotic and immunomodulatory properties, in addition to their ability to differentiate into several specialized cell lines. Cells can be carried through a biological delivery system, such as fibrin glue, which acts as a temporary matrix that favors cell-matrix interactions and allows local and paracrine functions of MSCs. Thus, the aim of this systematic review was to evaluate the potential of fibrin glue combined with MSCs in nerve regeneration. The bibliographic search was performed in the PubMed/MEDLINE, Web of Science and Embase databases, using the descriptors (“fibrin sealant” OR “fibrin glue”) AND “stem cells” AND “nerve regeneration”, considering articles published until 2021. To compose this review, 13 in vivo studies were selected, according to the eligibility criteria. MSCs favored axonal regeneration, remyelination of nerve fibers, as well as promoted an increase in the number of myelinated fibers, myelin sheath thickness, number of axons and expression of growth factors, with significant improvement in motor function recovery. This systematic review showed clear evidence that fibrin glue combined with MSCs has the potential to regenerate nervous system lesions.

## 1. Introduction

The nervous system is a highly specialized tissue, responsible for the functional sensory and motor activity of the organism. As other tissues, the nervous system is susceptible to trauma by mechanical, electrical and thermal action, as well as by ischemic compression or drug injection [1,2]. The severity of the sequelae and the tissue recovery ability depends on the nature and extent of the injury. Physiologically, the nervous system has a spontaneous self-repair capacity, with a regenerative rate of 1 to 3 mm per day [2]. However, adequate functional recovery of this tissue is still a major challenge, especially in large-scale injuries [2,3]. Thus, in most cases, therapeutic and surgical interventions are essential to encourage the regeneration of the nervous system [2,4]. Although, the late or inadequate interventions can cause several disorders, such as chronic pain, neuropathies, muscle atrophy, paralysis and severe functional incapacity with compromised quality of life [2,5].

Several strategies have been proposed to favor the regeneration of nervous tissue injuries, such as epineural suture, autogenous nerve grafts, allografts, nerve conduits, cell therapy and biological molecules, such as growth factors [5]. All these strategies have advantages and disadvantages, with limited potential to regenerate the damaged nerve [5]. Among the available techniques, autogenous nerve grafts are considered the gold standard, especially in cases of large nerve gaps [1,3,4,5,6,7,8,9]. Autogenous nerve grafts constitute a biological scaffold that contains native cells, neurotrophic growth factors, blood vessels, extracellular matrix proteins, adhesion molecules and neuronal cells, such as Schwann cells [5,6,7,8]. However, some disadvantages are associated with autogenous grafts, such as sensory-motor loss, morbidity, scarring and the possibility of neuroma formation in the donor area, in addition to the limited supply of donor nerve [4,7].

Allografts, in turn, provide availability of material and avoid the lack of donor área [5]. Despite the absence of viable cells, allografts maintain a native matrix that supports axonal growth, cell migration and angiogenesis [4,10]. Although, the required immunosuppression and inherent complications are disadvantages of allografts [3,4,7,8]. Nerve conduits are an option for the treatment of peripheral nerve injuries [5]. Conduits are tubular structures that surround the ends of the nerve and favor the direction of axonal growth [4]. The conduits can be constructed of synthetic materials, biodegradable or of natural components, such as veins, arteries and tendons. Nonetheless, an ideal conduit must have some fundamental properties, such as biocompatibility, biodegradability, low immunogenicity, mechanical strength and adequate permeability to allow the exchange of nutrients and biological molecules [1]. Additionally, the success of this technique depends on the formation of a fibrin matrix in the conduit lumen, which will support vascular infiltration and cell migration [5]. Furthermore, the use of conduits should only be indicated for the treatment of small nerve gaps (<3 cm) [1,3,4,5,7].

In addition to these strategies, cell therapy emerges as an interesting alternative for treating nervous tissue injuries. Cellular components play an important role in overall tissue healing.In the same way, nerve regeneration is a complex process that requires the interaction of various types of cells, extracellular matrix components, blood vessels, cytokines and growth factors [1]. Besides that, it is also critical that an endogenous fibrin matrix forms at the injury site in order to support axonal growth, vascular infiltration and immune system cell migration [4,8]. Nerve regeneration process involves a sequence of steps. After the injury, morphological and molecular changes occur in nerve cells, transport of proteins and influx of ions, such as calcium [4]. These events signal a disturbance in the homeostasis of the microenvironment that leads to changes in cell metabolism, with consequent activation of several signaling pathways and regulation of gene expression [4]. Schwann cells, macrophages and fibroblasts act in nerve regeneration from the initial stages. Schwann cells act together with macrophages in the Wallerian degeneration process, phagocytizing fragmented axons and myelin debris [4].

Throughout the regenerative process, the cells present in the microenvironment, mainly Schwann cells, overexpress neurotrophins and others growth factors involved with neuronal regeneration. Neurotrophins include nerve growth factor (NGF), brain derived neurotrophic factor (BDNF), neurotrophin-3 (NT-3) and neurotrophin-4/5 (NT-4/5), which are responsible for neuron growth and survival [8,11]. Other factors stimulate cell proliferation, differentiation and survival, such as ciliary neurotrophic factor (CNTF), glial cell line-derived growth factor (GDNF) and fibroblast growth factor (FGF) [8]. Among them, NGF is the most commonly characterized growth factor to assess the neurotrophic potential of cells [11]. Thus, nerve regeneration process is marked by overexpression of genes related to the inflammatory response, angiogenesis, synthesis of neurotrophic factors and expression of proteins involved in axonal growth and nerve fiber myelination [4]. Considering these issues, Schwann cells constitutes one of the most promising strategies to favor the regeneration of nervous tissue [5,12,13,14,15,16,17].

However, certain limitations make it difficult to use Schwann cells, such as limited availability of donor tissue and difficulties related to the cultivation and isolation of these cells [4,5]. As a result of these limitations, other cell lines have been investigated for use in regenerative therapy, such as embryonic stem cells (ESCs), induced pluripotent stem cells (iPSCs) and mesenchymal stem cells (MSCs) [5,12,17]. Among the candidate cells, MSCs stand out as a viable alternative for use in regenerative medicine, considering the ethical and genetic manipulation issues that limit the use of ESCs and iPSCs, respectively [12,17].

MSCs are undifferentiated cells that can be obtained from different sources, such as bone marrow, periosteum, adipose tissue, skin, muscle, tendons, umbilical cord, peripheral circulation and dental tissue [18,19]. Due to their adherence to plastic, MSCs can be expanded in culture and characterized by the expression of specific surface antigens, such as CD29, CD73, CD90 and CD109 [17,18,20,21,22]. MSCs have several biological properties that favor tissue regeneration. MSCs have the capacity for self-renewal, proliferation and differentiation in different cell lines, depending on the stimulus from the microenvironment [17,18,20,21]. Under neural induction, MSCs can differentiate into neuronal cells and express neurotrophic growth factors, such as nerve growth factor (NGF) and brain-derived neurotrophic factor (BDNF) [22,23,24,25,26]. MSCs also have anti-apoptotic, angiogenic and immunomodulatory potential, and act by regulating the production of inflammatory cytokines through several signaling pathways [19,20,21,25,27]. 

Modulation of the inflammatory response is essencial to limit tissue destruction, to reduce the formation of fibrous scars and to favor the regeneration of the injured area [12]. Additionally, MSCs express angiogenic factors, such as vascular endothelial growth factor (VEGF), which contribute to vascular neoformation [22,28,29]. MSCs are still able to migrate to the site of injury and recruit other cells through paracrine mechanisms [21].In nervous system injuries, MSCs can be used to favor axonal regeneration and myelinization of the myelin sheath [22,23,24,26,30]. Together, these characteristics make MSCs promising candidates for use in cell therapy strategies.

For use in cell therapy strategies, MSCs can be harvested from autologous tissues and implanted at the injury site through a cell delivery vehicle, such as fibrin glue or fibrin sealant. In regenerative medicine, fibrin glue can be used as a delivery system for drugs, biomolecules, growth factors and cells [31,32,33,34]. Fibrin is a natural polymer formed by the combination of thrombin and fibrinogen, which are components of the blood coagulation system [33,35,36]. Fibrin glue had its use approved by the FDA and since 1976 it has been widely used as a hemostatic agent to treat coagulopathies and in several surgical specialties, such as neurological, gastrointestinal and cardiovascular surgeries, among others [31,35,37]. In medical practice, fibrin glue can be obtained from autologous blood components, which reduces the risk of immune reactions [31,35,38]. 

Fibrin glue has several other interesting biological properties for use in regenerative therapies. Fibrin is a biocompatible matrix that can be naturally degraded by the action of fibrinolytic enzymes, such as metalloproteinases [33,39,40,41]. When used as a cell delivery vehicle, fibrin glue has the advantage of allowing a uniform distribution of cells in the matrix and it can also be injected into the lesion area through less invasive procedures [34,41]. Furthermore, fibrin glue provides a bioactive matrix that favors cell adhesion, viability, proliferation and differentiation [33,34,42]. The fibrin glue also contains numerous binding sites with cells, molecules and growth factors that act in the tissue regeneration process [34,41]. Thus, the fibrin glue favors cell-matrix interactions and supports axonal growth. Additionally, the porosity of the fibrin matrix favors angiogenesis and vascular infiltration, which are essential for the restoration of the injured area [34,41].

**Figure 1:**
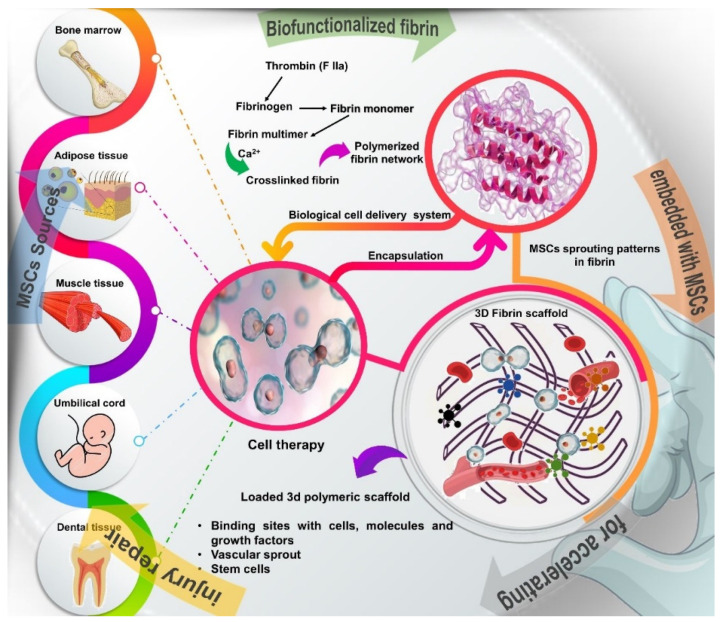
Schematic overview of the different sources of stem cells, such as bone marrow, adipose tissue, muscle, tendons, umbilical cord, and dental tissue and description of how to obtain the fibrin network, a natural polymer formed by the combination of thrombin and fibrinogen, which are components of the blood coagulation system. Fibrin glue provides a 3D bioactive matrix that favors cell adhesion, viability, proliferation and differentiation, in addition to containing numerous binding sites with cells, molecules and growth factors that act in the tissue regeneration process.

The viability of growing MSCs in fibrin glue has been reported in several studies [23,24,26,30,40,43]. Kalbermatten et al. (2008) reported that fibrin glue improved the adhesion of MSCs and Schwann cells in bioresorbable poly-3-hydroxybutyrate nerve conduits [43]. This study showed that cells seeded in fibrin glue were optimally distributed along the conduit, better than those seeded in growth medium [43]. In the study by Gardin et al. (2011), adult stem cells organized in neurospheres and seeded in fibrin glue meshes were able to grow and differentiate into glial/neuron cells under neural induction, without any chromosomal alteration [30]. Likewise, Park et al. (2012) showed that MSCs were able to differentiate into neuronal cells, to exhibit neuron-like cell morphology and to express various neural markers and transcription factors [23] (Figure 1).

Further studies also demonstrated that MSCs cultivated in fibrin glue were able to differentiate into neuronal cells and to express neurogenic marker proteins, such as microtubule-associated protein 2 (MAP2), nerve growth factor (NGF), brain-derived neurotrophic factor (BDNF) and Tau protein [24,26]. The compatibility and bioactivity of MSCs in fibrin glue was also reported by Krug et al. (2018) [40]. This study showed that the fibrin glue favored adhesion, migration, proliferation and metabolic activity of MSCs in culture [40]. These authors also reported that MSCs were uniformly distributed in the fibrin glue and strongly interacted with matrix components [40]. Another relevant data addressed in this study refers to the fact that, after 14 days of culture, fibrin degradation was accompanied by overexpression of metalloproteinases by MSCs, which could favor axonal growth and regeneration in clinical situations [40]. 

Thus, considering the several biological properties of MSCs and fibrin matrix, this systematic literature review aimed to investigate the potential of therapy with MSCs associated with fibrin glue on the regeneration of the central or peripheral nervous system.

## 2. Materials and Methods

### 2.1. Study Design and Bibliographic Search Strategy

This systematic review was conducted according to the PRISMA guidelines and the PICO strategy (Patient, Intervention, Comparison and Outcomes). The electronic bibliographic search was carried out in August–September/2021 in the PubMed/MEDLINE, Embase and Web of Science databases, combining the descriptors (“fibrin sealant” OR “fibrin glue”) AND “stem cells” AND “nerve regeneration”. The design of this review involved studies published up to the year 2021 that evaluated the effect of therapy with MSCs associated with fibrin glue on central and peripheral nervous system regeneration.

### 2.2. Study Eligibility

The eligibility criteria involved studies that compared the effect of fibrin glue alone or associated with MSCs as regenerative therapy for the treatment of nerve injuries, with or without the use of nerve conduits. Studies that used different analysis methods were considered. In vitro studies and literature reviews were not included within the eligibility criteria. Studies that used only differentiated or genetically modified MSCs to express growth factors were excluded from this review. Likewise, studies that evaluated the regenerative effect of therapy with MSCs and fibrin glue, through generalized exposure of experimental groups to immunosuppressive or others drugs, were also excluded.

For the eligibility of the experiments included in Tables 1 and 2 for detailed analysis, manuscripts with methodology and main results and their conclusions on neuroregeneration were also considered, in relation to morphophysiological and functional reparative aspects. Furthermore, the quality of the strategy for selecting studies included in this review was confirmed by the analysis of independent reviewers. The selection of studies was carried out carefully following the eligibility criteria in order to minimize bias.

## 3. Results

**Figure 2:**
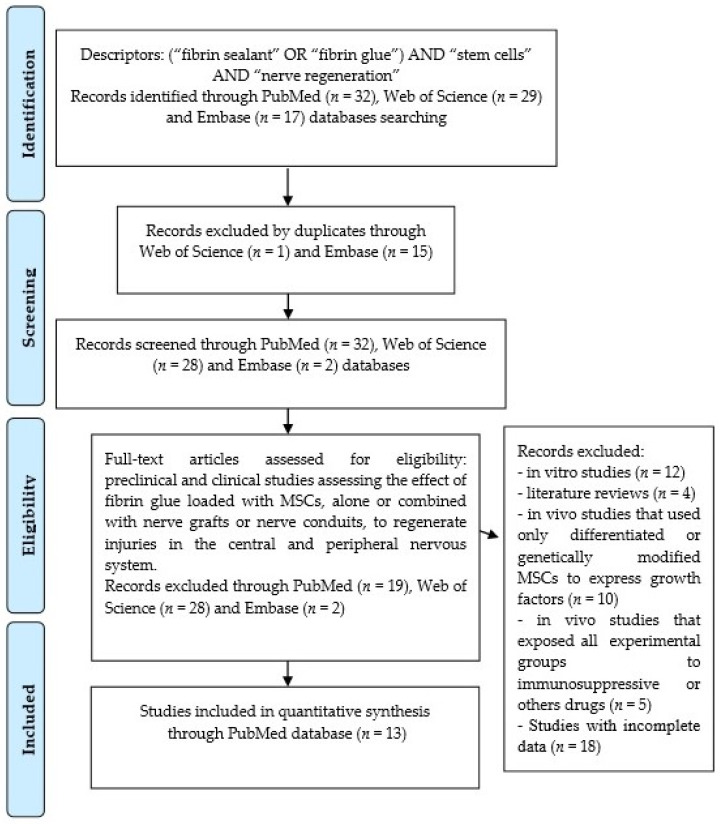
Prisma flow diagram resulted from an electronic bibliographic search for the potential of MSCs associated with fibrin glue in central and peripheral nervous system regeneration.

The results of the bibliographic research are outlined in the Prism Flow Diagram (Figure 2). The search strategy showed 32 articles in the PubMed/MEDLINE database, of which 19 were excluded because they were outside the eligibility criteria. In the Web of Science and Embase databases, 29 and 17 articles were found, respectively, which were not included due to duplicity or because they were outside the eligibility criteria. We selected 13 studies from the PubMed/MEDLINE database to compose this review.

The literature search strategy resulted in the compilation of studies with animal models that used MSCs and fibrin glue to treat peripheral nerve and spinal cord injuries. Table 1 and Table 2 summarizes the main results of the studies included in this review, containing in its columns the manuscript reference, the original source of the stem cells, the treatment groups used in each of the studies, the location of implantation of the stem cells, which were the analyzes performed in the experiment and, in the last column, the main results and conclusions. Table 1 shows the experiments with peripheral nerve injuries and Table 2 shows central nerve injuries.

In the studies included in this review, therapy with MSCs and fibrin glue was used to treat sciatic [24,26,44,45,46,47,48,49,50,51], femoral [23], mandibular [52] and spinal cord nerve injuries [53]. MSCs were harvested from several sources, such as bone marrow from the tibia/femurs [26,44,45], iliac crest [47], adipose tissue [46,51], aminiotic fluid [48,50,54], fetal brain [52,53], dental pulp [24] and skin [23]. Among studies, the effect of therapy on nerve regeneration was evaluated by one or more methods, such as physical examination, electromyography, electrophysiology, immunohistochemistry, immunofluorescence, enzyme-linked immunosorbent and tunnel assays, histology and morphometric analysis.

**Table 1:** Studies selected according to eligibility criteria, in peripheral nerve injuries, in chronological order.

Reference	Stem Cells Source	Treatment Groups Delivery System	Intervention Implantation Site	Analysis	Main Outcomes Conclusions
Pan et al. (2006) [50]	Allogeneic MSCs (amniotic fluid)	G1: Fibrin glue (FG) + Surgicel^®^ G2: FG + MSCs + Surgicel^®^	Rats sciatic nerve injuries were sutured, the gap (5 mm) was filled with MSCs + FG + Surgicel or FG + Surgicel (*n* = 10). Analyses were performed after 8 weeks of the procedures.	Electrophysiological and immunohistochemical analysis.	MSCs + FG + Surgicel^®^ significantly improved muscle action potential amplitude and axonal growth. Compound muscle action potential values for G1 and G2 were 28.5 ± 1.3% and 42.5 ± 1.25%, respectively. Amniotic MSCs significantly improved nerve regeneration in a sciatic nerve gap.
Pan et al. (2007) [48]	Allogeneic rats MSCs (amniotic fluid)	G1: Fibrin glue (FG) + Surgicel^®^ G2: FG + MSCs + Surgicel^®^	Rats sciatic nerve injuries (crush site) were sutured and wrapped with FG (*n* = 20) or FG + MSCs (*n* = 30). Analyzes were performed after 4 weeks of the procedures.	Enzyme-linked immunosorbent assay (ELISA), immunocytochemistry, motor function, electrophysiology, histology and immunocytochemistry	FG + MSCs significantly improved the motor function recovery, reduced the fibrosis at the injury site and favored the expression of neurotrophic factors, such as CNTF and NT-3.Compound muscle action potential values for G1 and G2 were 27.8 ± 4.22% and 67 ± 6.98%, with conduction latency of 3.91 ± 0.303 and 1.33 ± 0.048 msec, respectively. MSCs favored the sciatic nerve regeneration after crush injury.
Pan et al. (2009) [54]	Human amniotic fluid MSCs	G1: Fibrin glue (FG) + Surgicel^®^ G2: FG + Surgicel^®^ + Natto G3: MSCs + FG + Surgicel^®^ G4: MSCs + FG + Surgicel^®^ + Natto	Rats sciatic nerve injuries (crush site) were sutured and wrapped with FG or FG + MSCs (*n* = 6). G2/G4 were fed with Natto extract (16 mg/day) for 7 days. Analyzes were performed after 7 and 28 days of the procedures.	Electrophysiological, immunohistochemical, histological, cell apoptosis (Tunel assay) and pro-inflammatory cytokines analysis.	MSCs + FG + Surgicel^®^ promoted better nerve regeneration than the FG + Surgicel^®^ alone. Compound muscle action potential values for G1, G2, G3 and G4 were 0.25 ± 0.04%, 0.47 ± 0.03%, 0.51 ± 0.02% and 0.68 ± 0.02%, with conduction latency of 3.92 ± 0.31%, 1.85 ± 0.07%, 1.84 ± 0.08%, and 1.38 ± 0.11% respectively. Natto, alone or combined with MSCs, reduced cell apoptosis and proinflammatory cytokines levels, such as *TNF-α* and *IL-1β*. Combined treatment of MSCs+Natto showed the most beneficial effects.
McGrath et al. (2012) [47]	Human BM-MSCs (iliac crest)	G1:fibrin conduit (FC) + fibrin matrix (FG, Tisseel^®^) G2: FC + FG + cyclosporine G3: FC + FG + BM-MSCs G4: FC + FG + BM-MSCs + cyclosporine	Implantation of the fibrin conduit (14 mm) in rats sciatic nerve injuries (gap 10 mm). The conduct was sutured and filled with FG containing BM-MSCs. G2/G4 received daily intraperitoneally injections of cyclosporine A. Analyses were performed after 3 weeks of the procedures.	Immunohistochemistry	FC + FG + MSCs + cyclosporine was the most effective treatment to increase axonal regeneration and to reduce the macrophage-mediated inflammatory response.
Park et al. (2012) [23]	Autologous porcine SMSCs (ear skin)	G1: Collagen membrane (Lyoplant^®^) + fibrin glue (FG) + SMSCs G2: FG	Implantation of the collagen membrane in porcine femoral nerve injuries (gap 10 mm). The membrane was sutured and filled with FG containing SMSCs (*n* = 4). Analyses were performed after 2 and 4 weeks of the procedures.	Immunohistochemical and histological analysis.	G1 showed remarkable nerve regeneration with complete nerve bundles and higher expression of S-100 protein and p75 nerve growth factor. Autologous SMSCs and fibrin glue may act as an available substitute for nerve conduit material in peripheral nerve defect sites.
Zhao et al. (2014) [26]	Allogeneic rats BM-MSCs (femurs/tibias)	G1: Autograft G2: Acellular allograft (AL) + Fibrin glue (FG) + BM-MSCs (MSCs injected inside the graft) G3: AL + FG + BM-MSCs (MSCs injected around the graft) G4: AL + FG	Implantation of the graft in rats sciatic nerve injuries (gap 12 mm), suture and injection of FG containing BM-MSCs into/around the graft. Analyzes were performed after 2 weeks (*n* = 5) and 12 weeks (*n* = 8) of the procedures.	Muscle weight, histological, histomorphometric, sensory and motor functional analysis.	G1, G2 and G3 groups showed greater potential for nerve regeneration with greater axonal growth and myelination of nerve fibers. The graft implant with FG + BM-MSCs is successful in maintaining nerve structure and may support nerve regeneration.
Kurwale et al. (2015) [45]	Allogeneic rats BM-MNCs (femurs/tibias)	G1: Fibrin glue (FG) (Tisseel^®^) G2: FG + BM-MNCs	Rats sciatic nerve injuries were microsutured, the gap (2 mm) was filled with BM-MNCs and covered with FG (*n* = 5, per group/period). Analyses were performed after 15 and 60 days of the procedures.	Histological, immunohistochemical and morphometric analysis	FG + BM-MNCs showed a significant increase in axon diameter, nerve and myelin thickness at the repair site and at the distal most sites on early and late phases, respectively. Transplantation of BM-MNCs to the site of peripheral nerve injury leads to a significantly better recovery.
Reichenberger et al. (2015) [51]	Allogeneic rats AD-MSCs (inguinal tissue)	G1: Fibrin glue (FG) (Beriplast^®^) G2:FG + AD-MSCs	Sciatic nerve injuries of 50 rats were microsutured and the gap was filled with FG or FG containing AD-MSCs. Analyses were performed after 7, 21, 35 and 63 days of the procedures.	Immunofluorescence, muscle weight, histological and histomorphometric analysis.	FG + AD-MSCs showed better nerve regeneration with significant increase in muscle weight, axon number and myelin sheath thickness. AD-MSCs significantly enhanced the regeneration of peripheral nerve injuries after primary coaptation.
Ullah et al. (2017) [24]	Human dental pSCs (pulp-derived stem cells)	G1: Sham (*n* = 4) G2: Collagen membrane (Lyoplant^®^) + fibrin glue (FG) G3:Lyoplant^®^ + FG + pSCs G4:Lyoplant^®^ + FG + DpSCs	Implantation of the collagen membrane in rats sciatic nerve injuries (gap 5 mm). The membrane was sutured and filled with FG containing pSCs or differentiated neuronal cells (DpSCs) (*n* = 6/per group). Analyzes were performed after 12 weeks of the procedures.	Muscle contraction activity, immunohistochemical and histological analysis.	G3 and G4 showed considerable and similar regenerative potential, better muscle activity and greater expression of specific markers for angiogenesis, axonal fiber and myelin sheath, such as VEGFR-1, GFAP, S-100 protein, MBP-2 and p75NGFR. pSCs could exhibit excellent peripheral nerve regeneration potential.
Goel et al. (2019) [44]	Allogeneic rats BM-MNCs (femurs/tibias)	G1: Fibrin glue (FG) (Tisseel^®^) G2:FG + BM-MNCs	Rats’ sciatic nerve injuries were microsutured, filled with BM-MNCs and covered with FG (*n* = 5, per group/period). Analyses were performed after 30 and 60 days of the procedures.	Histological analysis	FG + BM-MNCs presented better axonal regeneration and remyelination with greater density of myelinated fibers. Local delivery of BM-MNCs improved peripheral nerve regeneration.
Masgutov et al. (2019) [46]	Allogeneic rats AD-MSCs (inguinal tissue)	G1:autologous nerve graft (AG) + fibrin glue (FG) + AD-MSCs Tissucol-Kit^®^ G2: AG + FG G3: AG G4: Intact animals	Rats’ sciatic nerve injuries (gap 5 mm) were filled with AG, sutured and covered with FG containing AD-MSCs (*n* = 5). Analyses were performed after 30 and 60 days of the procedures.	Functional motor test, electromyography, EasyLDI laser Doppler and morphological analysis	AG + FG + AD-MSCs increased the number of myelinated fibers and neurons of the L5 spinal ganglion, with improved motor activity and angiogenesis. AG + FG + AD-MSCs resulted in motor function recovery after injury.
Bayir et al. (2021) [52]	Allogeneic rats NMSCs (fetal brain)	G1: No treatment G2: NMSCs G3: Fibrin glue (FG) Tisseel^®^ G4: NMSCs + FG	Injection of NMSCs or NMSCs + FG in rats mandibular facial nerve injuries(crush). Analyses were performed immediately after surgery and after 3, 5 and 8 weeks of the procedures.	Physical examination, TUNEL assay and histochemical analysis.	NMSCs + FG showed a statistically significant functional improvement. NMSCs + FG may play a promising role as adjuvant regenerative therapy in traumatic facial paralysis.

**Table 2:** Studies selected according to eligibility criteria, in central nerve injuries.

Reference	Stem Cells Source	Treatment Groups Delivery System	Intervention Implantation Site	Analysis	Main Outcomes Conclusions
Pan et al. (2008) [53]	Allogeneic rats NSCs (fetal brain)	G1: Fibrin glue (FG) G2: FG + granulocyte colony-stimulating factor (G-CSF) G3: FG + NSCs G4: FG + NSCs + G-CSF	Injection of NSCs into the rats spinal cord (T8-T9) gap (2 mm) and sealing with FG + gelfoam. Subcutaneous injection of *G-CSF* for 5 days in G2/G3 groups (*n* = 10). Analyses were performed after 3 months of the procedures.	Electrophysiological, hind-limb motor function, histological and immunohistochemical analysis.	FG + NSCs + G-CSF significantly improved clinical motor activity, conduction latency and spinal cord regeneration scores. Motor evoked potential values for G1, G2, G3 and G4 were 24.69 ± 3.51, 31.64 ± 3.06, 38.97 ± 2.30 and 47.7 ± 3.17 mV, with conduction latency of 1.54 ± 0.04, 1.39 ± 0.03, 1.39 ± 0.04 and 1.29 ± 0.02 msec, respectively. Therapy associated with NSCs and C-CSF promoted better spinal cord regeneration.

Overall, the studies showed that fibrin glue combined with MSCs significantly favored nerve regeneration when compared to the isolated use of fibrin glue. Therapy with MSCs favored axonal regeneration and remyelination of nerve fibers, as well as increased myelinated fiber thickness, axon number and expression of neurotrophic factors. Additionally, treatment with MSCs improved muscle weight and motor function recovery, while reducing fibrosis at the injury site. In the study by Pan et al. (2006), treatment with fibrin glue and MSCs significantly improved the compound muscle action potential (42.5 ± 1.25%) compared to the isolated use of fibrin glue (28.5 ± 1.3%) [50]. Likewise, Pan et al. (2007) reported that groups treated with fibrin glue alone or associated with MSCs presented compound muscle action potential of 27.8 ± 4.22% and 67 ± 6.98%, with conduction latency of 3.91 ± 0.303 and 1.33 ± 0.048 msec, respectively [48]. When used with autologous nerve graft, fibrin glue with adipose MSCs (AD-MSCs) promoted a significant increase in the number of myelinated fibers with improved motor activity and angiogenesis, compared to the use of autologous graft alone or associated with fibrin glue without cells [46].

Likewise, the use of acellular allograft associated with fibrin glue and bone marrow MSCs (BM-MSCs) presented regenerative potential similar to the isolated use of autologous graft and superior to the use of allograft combined with fibrin glue without cells [26]. Studies that administered agents with anti-inflammatory or immunomodulatory properties, such as Natto extract [54], granulocyte colony-stimulating factor (G-CSF) [53] and cyclosporine A [47], concurrently with therapy with fibrin glue and MSCs, reported an additional beneficial effect on certain parameters. These studies showed a reduction in cell apoptosis, in the inflammatory response mediated by macrophages and in the levels of pro-inflammatory cytokines, such as TNF-α and IL-1β, in addition to an improvement in axonal regeneration and functional motor activity.

Nerve regeneration was also achieved in studies that enveloped the resected nerve ends with absorbable bovine collagen dura mater (Lyoplant^®^) [23,24] or fibrin conduit [47] and implanted fibrin glue with MSCs into the biodegradable nerve tubule. Finally, the study that compared the effect of implantation of fibrin glue containing undifferentiated or differentiated MSCs in neuronal cells related that nerve regeneration was not affected by the cellular differentiation stage, as the two types of cells presented considerable and similar regenerative potential [24], exhibiting greater expression of specific markers for angiogenesis, axonal fiber and myelin sheath, such as vascular endothelial growth factor receptor-1 (VEGFR-1), glial fibrillary acid protein (GFAP), S-100 protein, myelin basic protein-2 (MBP-2) and p75 nerve growth factor receptor (p75NGFR).

## 4. Discussion

The studies included in this systematic review used animal models to assess the regenerative potential of therapy with fibrin glue and MSCs in the treatment of nervous system injuries. Of the studies included in this review, six of them compared the effect of fibrin glue alone or associated with MSCs to treat sciatic [44,45,48,50,51] and mandibular nerve injuries [52]. The outcomes of these studies showed that the association of fibrin glue with MSCs significantly favored axonal regeneration and myelination of nerve fibers compared to the use of fibrin glue without cells [44,45,48,50,51,52]. Additionally, treatment with fibrin glue and MSCs resulted in a significant increase in the levels of neurotrophic factors [48] and less formation of scar tissue at the site of nerve injury [46,48], which accelerated reinnervation and recovery of functional activity [46]. Alterations in the levels of neurotrophic growth factors are indicators of nerve regeneration process, since low levels of these factors are found in intact peripheral nerves, whereas both the expression of mRNA and the synthesis of the corresponding proteins significantly increase when the nerve is transsected or crushed [55].

In the study by Pan et al. (2007), ELISA measurement and immunocytochemical analyzes revealed increased levels of ciliary neurotrophic factor (CNTF) and neurotrophin-3 (NT-3) in the regenerating sciatic nerve and positive staining for these factors in transplanted MSCs, respectively [48]. Kurwale et al. (2015) detected changes in the microstructure of the injured sciatic nerve in the early postoperative phase (15 days), so that treatment with fibrin glue and MSCs promoted a significant increase in the mean diameter of the axon, in the mean nerve thickness and in the myelin thickness at the repair site compared to the isolated use of fibrin glue [45]. Similar results were obtained by Goel et al. (2019), who reported that sciatic nerve regeneration was more significant in the group treated with fibrin glue and MSCs at 30 and 60 days after surgery, while degenerative changes, such as ballooning of axons and degeneration of the sheath of myelin, were more prominent in the group treated only with fibrin glue [44]. 

Nervous system regeneration is a complex biological process and the isolated use of fibrin glue is not sufficient to promote significant regeneration. The regenerative process takes place through the interaction of cells, matrix components, growth factors and cytokines. When implanted at the site of nerve injury, MSCs are able to differentiate into neuronal cells and express neurotrophic factors, which are essential for axonal growth and nerve fiber remyelination [23,24,26]. In addition, MSCs express angiogenic factors and several other growth factors that, through various signaling pathways, act on cells present in the tissue, such as fibroblasts, macrophages and endothelial cells, modulating the inflammatory response, reducing scar formation and favoring vascularization of the regenerating nerve [56,57]. 

It is important to emphasize, however, that an adequate regeneration of the nervous system depends on the correct growth orientation of the injured axons. Thus, analyzses of postoperative motor function are important to assess whether there was an adequate regeneration of the injured nerve extremities [55]. Considering these issues, in addition to histological analyses, some studies performed physical [52], electrophysiological [48,50] and muscle weight [51] examinations to analyze the recovery of sensory and motor functions of the injured nerve. These analyses showed that therapy with fibrin glue and MSCs significantly improved functional activity [52], muscle action potential amplitude and motor function recovery [48,50], with significant increase in muscle weight [51] and satisfactory nerve regeneration even in gap of 5 mm [50]. 

In the study by Pan et al. (2006), electrophysiological analyzes showed that the group treated with fibrin glue and MSCs had an average action potential of 42.5%, differing statistically from the group treated only with fibrin glue (28.5%) [50]. The outcomes of these studies showed that the interactions that MSCs established in the damaged tissue microenvironment contributed not only to favor axonal growth, but to modulate the inflammatory response and reduce the formation of scar tissue, facilitating the reorganization of nerve fibers and orientation of axonal growth. Another relevant issue is that, in these studies, therapy with fibrin glue and MSCs had the potential to regenerate both crush and transection injuries, whereas axons regenerate and interconnect more accurately in crush injuries, in which often Schwann cells are maintained viable, rather than by transection lesions [55].

The regenerative potential of MSCs was also observed in studies that used nerve conduits to guide axonal growth [23,24,47]. The implantation of conduits that act as guide tubules is a strategy used to support and adequately direct axonal growth in peripheral nerve injuries [56]. In transsection injuries in which the nerve fiber is disruption, the use of conduits favors the correct orientation of growth between the extremities of the injured nerve, in addition to reducing fibroblast infiltration and the formation of scar tissue [56]. The success of this technique can be improved by filling the conduit with a bioactive matrix that provides binding sites with cells, molecules and growth factors, considering that nerve regeneration requires the formation of an endogenous supporting matrix [56]. In the three studies included in this review that used nerve conduits, the implant of fibrin glue in the conduit achieved even better results when associated with MSCs [23,24,47].

Thus, McGrath et al. (2012) obtained better axonal regeneration of the sciatic nerve in the groups treated with MSCs [47]. Corroborating these data, Ullah et al. (2017) obtained better muscle activity, greater expression of specific markers for angiogenesis, axonal fiber and myelin sheath in groups treated with MSCs associated with fibrin glue to regenerate sciatic nerve [24]. Additionally, this study also evaluated the rate of cellular apoptosis at the site of injury and found that fibrin glue minimized the effect of cytokines on implanted MSCs, keeping these cells viable in the damaged area [24]. Despite the inherent regenerative properties of MSCs, fibrin glue also played an important role in the tissue regeneration process, providing a bioactive matrix that supported the MSCs to perform their functions. Thus, these studies showed that fibrin glue loaded with MSCs can act as a biological substitute for filling nerve conduits in peripheral nerve injury regeneration strategies [23]. In addition to the filling components, the biological composition of the conduit is one of the factors that influence the success of this technique. 

Some of the studies in this review used biodegradable bovine collagen duramater (Lyoplant^®^) filled with fibrin glue and MSCs to regenerate gaps of 5 mm [24] and 10 mm [23] in the sciatic and femoral nerves, respectively. After 4 weeks, histological and immunohistochemical analyses showed remarkable nerve regeneration with complete nerve bundles in gaps of 10 mm with higher expression of S-100 protein and p75 nerve growth factor (p75NGFR) [23]. Collagen conduits allow the transport of nutrients and growth factors to the area of injury, favoring nerve regeneration. In addition to being semipermeable, collagen is a natural component of the extracellular matrix, which favors cell proliferation and migration [1,56]. Similar outcomes were also obtained by McGrath et al. (2012), who used fibrin conduit filled with fibrin glue and MSCs to regenerate nerve injury with a gap of 10 mm [47]. Like collagen, fibrin is an excellent biomaterial capable of supporting axonal growth, in addition to having a porous structure that allows the passage of molecules into the conduit [11,22,56]. Additionally, fibrin reduces the formation of scar tissue, favoring nerve regeneration [11,56]. 

However, although the use of biocompatible and biodegradable conduits constitutes an interesting strategy to direct growth and favor axonal regeneration, autogenous grafts are still the gold standard, especially in lesions with extensive loss of nervous tissue, with gaps greater than 3 cm [57]. Two studies included in this review investigated the regenerative potential of fibrin glue and MSCs associated with allograft or autologous nerve graft [26,46]. The study by Masgutov et al. (2019) used autologous nerve graft, fibrin glue and MSCs to treat a gap of 5 mm in a sciatic peripheral nerve [46]. In this study, the use of autologous nerve graft associated with fibrin glue and MSCs (AG + FG + AD-MSCs) resulted in better motor function recovery after injury compared to the use of autologous nerve graft isolated (AG) or combined with fibrin glue (AG + FG) [46]. Sixty days after injury, AG + FG + AD-MSCs showed a significant increase of 26% and 28% in motor activity when compared to the AG and AG + FG groups, respectively [46]. Additionally, AG + FG + AD-MSCs promoted an increase in the number of myelinated fibers and improved angiogenesis [46]. Laser Doppler analysis also showed that the vascular supply at the lesion site was reestablished after 14 days post-operatively in the AG + FG + AD-MSCs group, at levels similar to those found in intact animals [46]. However, no vascular difference between the different groups was found after 30 days [46]. Additionally, after 30 days, AG + FG + AD-MSCs showed a significant increase around 18% in the number of myelinated fibers in the distal segment of the nerve compared to AG + FG [46]. Both groups showed an additional increase of 20% in the number of myelinated fibers after 60 days postoperatively [46]. 

Corroborating these data, Zhao et al. (2014) showed that the allogeneic graft associated with fibrin glue and MSCs had a similar potential to the isolated use of autogenous graft to regenerate a gap of 12 mm in the peripheral sciatic nerve [26]. In this study, three months after the injury, both groups had greater regenerative potential with greater axonal growth and myelination of nerve fibers [26].

Some studies in this review investigated whether the regenerative potential of fibrin glue and MSCs could be improved by administration of drugs or extracts with an immunoregulatory or anti-inflammatory effects [47,49,53]. Pan et al. (2008) used harvested fetal brain stem cells (NSCs) to regenerate a gap of 2 mm in the spinal cord [53]. The cells were implanted and the lesion area was sealed with fibrin glue (FG) and gelfoam [53]. Two experimental groups also received subcutaneous injection of granulocyte colony-stimulating factor (G-CSF) for 5 days [53]. G-CSF has immunoregulatory, anti-inflammatory and anti-apoptotic effects on neuronal cells. Three months after the injury, the analyses showed that the therapy associating FG + NSCs + G-CSF promoted better spinal cord regeneration and improved clinical motor activity, conduction latency and spinal cord regeneration scores [53]. 

Posteriorly, these authors investigated the effects of Natto extract administration on the regenerative potential of therapy with fibrin glue and MSCs (FG + MSCs + Surgicel^®^) to treat sciatic nerve crush and showed that, 28 days after the injury, FG + MSCs + Surgicel^®^ promoted better nerve regeneration than the FG + Surgicel^®^ alone [49]. Additionally, animals fed with Natto extract had a lower rate of cellular apoptosis and lower levels of pro-inflammatory cytokines at the site of injury [49]. Nerve crush injury induces an increase in the number of vacuoles and a reduction in the expression of the S-100 protein, which is related to the nerve fiber myelination process [49]. Thus, the therapy combined with Natto extract showed other additional benefits, reducing the number of vacuoles at the injury site and increasing the expression of S-100 protein [49]. 

The study by McGrath et al. (2012) that used fibrin conduit and implantation of fibrin glue and MSCs to regenerate a gap of 10 mm in the sciatic nerve obtained better results in the groups in which intraperitoneal injections of cyclosporin A were administered [47]. Cyclosporine A is an immunosuppressive drug that has neuroprotective potential. The groups treated with FG + MSCs + cyclosporine showed better axon regeneration with reduced inflammatory response mediated by macrophages [47]. Cyclosporin A regulates the expression of matrix metalloproteinases, which are responsible for accelerating the degradation of the fibrin matrix. Thus, groups treated with cyclosporine showed faster degradation of fibrin glue and less macrophage infiltration [47]. The administration of drugs as a complementary therapy to surgical interventions for the treatment of injuries in the nervous system can have beneficial effects. However, the risks-benefits and possible side effects associated with drug therapies must be considered, especially with regard to the use of immunosuppressants [4,5]. Furthermore, research must evolve to assess the effect and safety of drug therapies, considering that there is a lack of clinical evidence about the best therapy to treat injuries in the nervous system [5]. 

**Figure 3:**
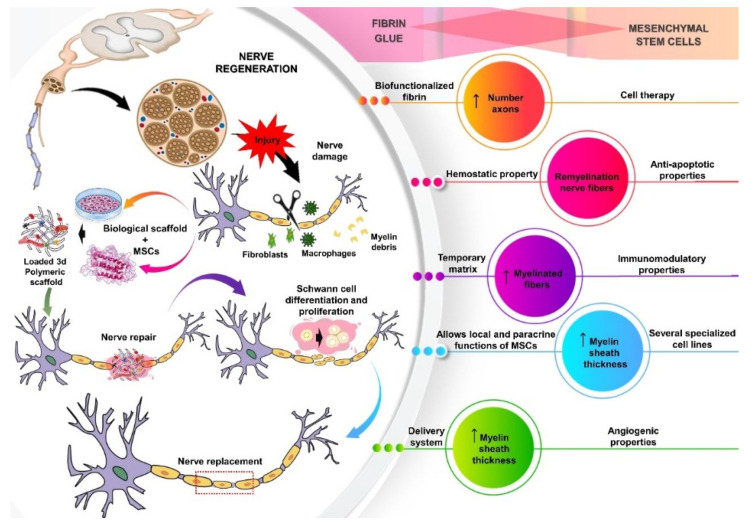
Schematic illustration of central or peripheral nervous system regeneration through cell therapy associated with a 3D polymeric matrix of fibrin. Furthermore, it explains the intrinsic characteristics of each, a proportionally synergistic effect when associated. The mesenchymal stem has angiogenic, anti-apoptotic and immunomodulatory properties, in addition to its ability to differentiate into several specialized cell lines. Fibrin glue has hemostatic properties and acts as a temporary matrix that favors cell-matrix interactions and allows local and paracrine functions of MSCs. In the nervous repair process, events occur that signal a disturbance in the homeostasis of the microenvironment, which leads to changes in cell metabolism, with consequent activation of several signaling pathways and regulation of gene expression. At this stage, the MSCs differentiate into Schwann cells, they overexpress neurotrophins and other growth factors involved in neuronal regeneration and the binding sites in the fibrin network provide interaction with macrophages and fibroblasts, aiding Wallerian degeneration, phagocytizing fragmented axons and myelin debris and sequentially regeneration nervous.

Regardless of the technique used, the main outcomes of these in vivo studies showed that therapy with fibrin glue and MSCs had considerable potential to regenerate lesions in the nervous system. The implantation of fibrin glue and MSCs reduced degenerative alterations, macrophage infiltration, destruction of myelin fibers and necrosis of small blood vessels [46]. Regarding MSCs, some important issues must be addressed, such as the tissue of origin and the stage of cell differentiation. In the studies included in this review, MSCs were harvested from different tissues, such as bone marrow, adipose tissue, amniotic fluid, fetal brain, skin and dental pulp. It is important to report that MSCs tend to keep peculiar characteristics of their tissue of origin, which can influence their regenerative properties [18,58,59] (Figure 3).

However, considering the main outcomes of the studies in this review, there was no deficit in terms of nerve tissue regeneration resulting from differences between the MSCs obtained from different sources. Regarding the stage of cell differentiation, only one of the studies included in this review compared the regenerative potential of undifferentiated MSCs and MSCs differentiated in neuronal cells [24]. In this study, the two groups of cells showed considerable and similar potential to regenerate sciatic nerve damage. These cells showed greater expression of specific markers for angiogenesis, axonal fiber and myelin sheath, in addition to better muscle activity, compared to the group treated only with collagen membrane and fibrin glue. Corroborating these data, the regenerative potential of MSCs differentiated into neuronal cells has been reported in several studies with animal models [13,14,15,16]. Therefore, there is evidence in the literature that therapy with MSCs has the potential to favor nerve regeneration, regardless of the stage of cell differentiation [60]. Some review studies have demonstrated the potential of stem cells in different areas of neuroregeneration, for example, in the treatment of traumatic brain injury [61,62].

Mesenchymal/stromal stem cells (MSCs) have translational potential in regenerative medicine, moving from researchers’ benches to clinical application, and have aroused interest as cell-based therapies for a variety of autoimmune and inflammatory diseases [63]. Furthermore, MSCs are environmentally responsive and they are able to adapt their behavior according to tissue challenges [64].

In summary, the methods of analysis of the different studies in this review provided relevant information about the effect of therapy with fibrin glue and MSCs, not only with regard to the histological aspects of the treated area, but also concerning the quality of the regeneration of the injured nerve, evaluated by the recovery of functional motor activity. In these studies, additional assays provided data about the alterations in the levels of neurotrophic growth factors, expression of specific markers of angiogenesis and pro-inflammatory cytokines at the site of injury. Taken together, these results showed that therapy with fibrin glue and MSCs had considerable regenerative potential and could be an advantageous strategy for treating nervous system injuries. Despite the promising results of these in vivo studies, research must advance in the field of clinical trials in order to assess the potential of cell-based therapy to regenerate nerve lesions with different patterns of severity.

## 5. Conclusions

The combined use of fibrin glue and MSCs has been used as a less invasive strategy to regenerate lesions in different types of tissues. In the nervous system, fibrin glue loaded with MSCs had significant potential to regenerate transection or crush injuries in peripheral nerves or spinal cord. Therapy with fibrin glue and MSCs favored axonal regeneration and remyelination of nerve fibers, as well as increased the number of myelinated fibers, myelin sheath thickness, number of axons and the expression of neurotrophic and angiogenic factors, with improved motor function recovery. Fibrin glue and MSCs associated with nerve grafts or immunoregulatory/anti-inflammatory drug therapies showed an additional beneficial effect on nerve regeneration. Likewise, the use of nerve conduits with implant of fibrin glue and MSCs also had considerable potential to regenerate peripheral nerve injuries.

In short, this systematic review based on in vivo studies showed clear evidence that therapy with fibrin glue and MSCs has the potential to regenerate nervous system damage. However, advances in research are still required to investigate the clinical efficacy of therapy based on combined use of fibrin glue and MSCs as a strategy for treating nervous system injuries, taking into account the regeneration of the damaged area and the recovery of functional motor activity.

## Data Availability

Not applicable.
